# Swing Regulates Movement Direction in the Sea Cucumber *Apostichopus japonicus* in the Presence of Food Cue: New Insights into Movement Patterns

**DOI:** 10.3390/ani13213388

**Published:** 2023-11-01

**Authors:** Zihe Zhao, Jiangnan Sun, Yushi Yu, Peng Ding, Jun Ding, Yaqing Chang, Chong Zhao

**Affiliations:** Key Laboratory of Mariculture & Stock Enhancement in North China’s Sea, Ministry of Agriculture and Rural Affairs, Dalian Ocean University, Dalian 116023, China; makaozhao@gmail.com (Z.Z.); jnsun77@gmail.com (J.S.); yushi.yu830@gmail.com (Y.Y.); 15179420083dp@gmail.com (P.D.); dingjun1119@dlou.edu.cn (J.D.); changlab456@gmail.com (Y.C.)

**Keywords:** sea cucumber, organisms without eyes, direction movement, swing state

## Abstract

**Simple Summary:**

The present study investigated the movement patterns of the sea cucumber *Apostichopus japonicus*. We found that the swing was an important state in the motion mode of sea cucumbers to regulate the movement direction, and the motion state did not change the direction of movement. In addition, food cues caused changes in the movement state of sea cucumbers, and the movement in the presence of food was directed rather than random and significantly affected swaying behavior. These novel findings provide new insights into the regulation of movement direction in eyeless organisms.

**Abstract:**

Regulating movement direction is essential in the locomotion of animals. Sea cucumbers, as eyeless animals, do not have eyes for the perception of the surrounding environment and food. They have a unique way of swinging their bodies when a food cue is detected, although they lack an important perceptual tool. The present study investigated the movement patterns of the sea cucumber *Apostichopus japonicus* in the absence of a food cue (experiment 1) and in the presence of a food cue (experiment 2). In experiment 1, we found that the movement of sea cucumbers was close to a linear motion (motion linearity 0.91 ± 0.01). In experiment 2, sea cucumbers most frequently adjusted the movement direction when being exposed to food (84 times/216 min), indicating that sea cucumbers adjusted the direction of movement in the swing state but not the motion state. In experiment 2, we found significantly lower time in the immobility state in the sea cucumbers in the presence of food cues compared to that of those without being exposed to food cues, and the frequency of the motion state in response to food cues was 1.6 times than that of those without food cue. This suggests that food cues cause the change in motion state in sea cucumbers. Swing frequency was 1.7 times higher in sea cucumbers exposed to food cues than that of those not exposed to food cues. Further, sea cucumbers in the presence of food showed significantly better performances in swing angle and swing velocity compared to those not exposed to food cues. This suggests that food cue significantly affects the swing state of sea cucumbers. Notably, the present study described the movement patterns of sea cucumbers when they detected food cues, and other factors (such as the detection of predators) need to be further studied. The present study provides new insights into the regulation of movement direction in eyeless organisms.

## 1. Introduction

The sense of direction is a basic cognitive function for the survival of animals [1,2]. The eyes are an important sensory organ in the animal kingdom as a result of evolution. Animals with eyes can accurately perceive space, locate in the environment, chase prey, and avoid obstacles [3]. Therefore, visual perception is important for animals to regulate the direction of their movements [4,5]. Water birds rely on vision to adjust the direction of body movement to catch prey [6], and salamander *Euproctus asper* shows a directed, visually dominated behavior [7]. Some animals are still able to move in complex environments, although they have not developed eyes due to environmental factors. This is because, for eyeless animals, the surrounding environment is perceived by organs outside the eyes. For example, brittle star *Ophiocoma wendtii* has a skeleton with crystals that function as a visual system [8], sea cucumbers use their tentacles and tube feet to find food or avoid moving toward bright light [9,10], sea urchins use extraocular vision to food and locate shelter [11], and salamander *Proteus anguinus* show well-developed chemo- and mechanosensory apparatus to catch prey [7]. Therefore, there are various ways to regulate the direction of movement for eyeless animals.

Sea cucumbers are typical eyeless animals that rely on various tentacle forms to extend their tentacles over the sediment surface, pick up sediment (deposit feeders), or feed in the water (suspension feeders) [12]. The aquaculture production of sea cucumbers has traditionally focused on deposit-feeding species [13,14], for example, *Apostichopus japonicus*. Their crawling posture resembles that of inchworms [15]. However, sea cucumbers have a small range of movement, and their movement trajectory can turn in different directions [16]. It is necessary to quantitatively describe the movement pattern and to reveal whether sea cucumbers actively regulate the direction of movement, although movement direction is unpredictable. Crawling behavior, which is initiated by a peristaltic wave moving forward along the body, is the most obvious behavior in the movement pattern of sea cucumbers [17,18,19]. In addition, prolonged immobility is another common phenomenon [20,21]. When the sea cucumber stays still, the front body occasionally rises and swings frequently from side to side [22], indicating an important exploration activity [23]. Therefore, the present study divided the motion mode of sea cucumbers into immobility state, motion state, and swing state, and explored whether these three states participated in or manipulated the regulation of the movement direction of sea cucumbers.

Although sea cucumbers do not have discrete visual organisms, they display goal-directed movement [24,25]. Therefore, sea cucumber is a good research model to study the motion direction regulation in goal-directed movement in eyeless animals. Sea cucumbers live in a complex environment, and their movement patterns are affected by many factors. For example, they have an escape response when face to predators [26]. Food is also an important factor affecting the movement of sea cucumbers, and the random walk models of sea cucumbers show greater directional movement in the presence of food [27]. Therefore, it is speculated that food cues cause changes in locomotion behavior, making sea cucumbers react differently in the presence and absence of food. Further, as an important exploratory activity, swing behavior is also one state of movement patterns of sea cucumbers. But, whether it is affected by the presence of food cues is not clear. Therefore, in order to investigate the effect of food on the movement and swing behavior, the present study compared the movement patterns of sea cucumbers in the presence and absence of food cues.

By tracking the movement direction and quantifying the movement pattern of sea cucumbers, this study aims to investigate the following: (1) how the movement direction of sea cucumbers is regulated; (2) characteristics of movement patterns in the foraging behavior of sea cucumbers; (3) swing behavior characteristics in foraging behavior of sea cucumbers.

## 2. Materials and Methods

### 2.1. Sea Cucumbers

The sea cucumbers *A. japonicus* (~15 g of wet body weight) were transported from a local hatchery to the Key Laboratory of Mariculture and Stock Enhancement in North China’s Sea, Ministry of Agriculture and Rural Affairs at Dalian Ocean University (121° 37′ E, 38° 87′ N). Sea cucumbers were kept in a 500 L tank at 12 ± 0.5 °C and fed with a mixture of fresh seaweed powder and sea mud (1:4) [28] until the experiments started. 

### 2.2. Experimental Design

#### 2.2.1. Experiment 1: Movement Pattern of *A. japonicus* without Food Cue

**Figure 1:**
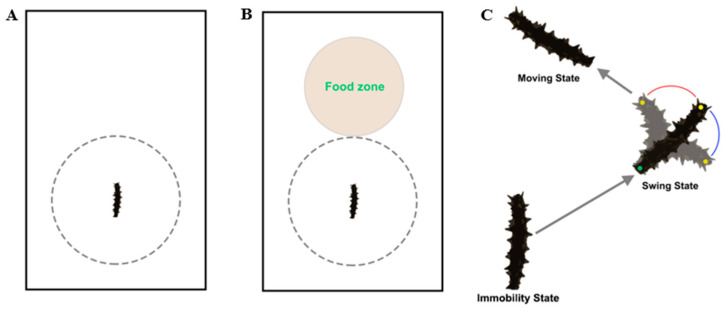
Schematic diagrams for experiments 1 (**A**) and 2 (**B**). A schematic representation of the path and three states of sea cucumbers (**C**).

To investigate the movement pattern of sea cucumber, we tracked the movement of *A. japonicus* in the experimental tank without substrate or water flow (length × width × height: 660 × 410 × 250 mm, Figure 1A). The sea cucumber was gently placed in the experimental tank. A camera (Legria HF20; Canon, Tokyo, Japan) above the experimental tank recorded the movement of sea cucumbers, and the recording stopped when the sea cucumber reached the gray line (Figure 1A). Each experiment was repeated 10 times using different sea cucumbers (N = 10). Seawater was changed for each test to avoid potential non-experimental impacts.

We divided the movement pattern of sea cucumbers into three states: immobility, motion, and swing (Figure 1C). The duration and frequency of three states were recorded, and each state was taken as a sample for data analysis. Movement speed (*v*) and displacement (*d*) of sea cucumber during three states were calculated as follows [29]:

(1) $$v=\sqrt{{\left[x_{i}\left(t\right)-x_{i}\left(t-5\right)\right]}^{2}+{\left[y_{i}\left(t\right)-y_{i}\left(t-5\right)\right]}^{2}}\times k/T$$

(2) $$d=\sqrt{{\left[x_{i}\left(t_{0}\right)-x_{i}\left(t_{e}\right)\right]}^{2}+{\left[y_{i}\left(t_{0}\right)-y_{i}\left(t_{e}\right)\right]}^{2}}\times k$$

where *x_i_*(*t*) and *y_i_*(*t*) are the coordinates of the animal *i* at minute *t*, *x_i_*(*t*_0_) and *y_i_*(*t*_0_) are the beginning coordinates of state, *x_i_*(*t_e_*) and *y_i_*(*t_e_*) is the end coordinates, *k* is the scale of the picture, and *T* is the time at which the state tracking ends.

The front body of sea cucumber was marked with yellow dot, and the rear body of sea cucumber was marked with green dot (Figure 1C). The swing angle *R* and swing speed (*v_s_*) was calculated as follows [30]:

(3) $$R=Acos\left(\frac{\vec{a}\times \vec{b}}{\left|\vec{a}\right|\times \left|\vec{b}\right|}\right)\times \frac{180}{\pi }$$

(4) $$v_{s}=R/T$$

where $\vec{a}$ is the vector of the sea cucumber at the beginning of the swing state, $\vec{b}$ is the end vector of the sea cucumber, and *T* is the time at which the swing state ends.

The linearity of sea cucumber movement was measured using the ratio of displacement to distance. The direction deviation angle during the motion state was represented as the angle between the body vector of sea cucumbers and the displacement vector of the motion state. The direction deviation before and after the swing state is represented as the angle between the displacement vectors of the two motion states.

#### 2.2.2. Experiment 2: Movement Pattern of *A. japonicus* in Response to Food Cue

To further investigate the foraging movement pattern of sea cucumber *A. japonicus*, we tracked the movement of sea cucumbers towards the food zone (Figure 1B), comparing it with sea cucumbers of experiment 1. The food zone was evenly spread with 20 g of kelp powder. For experiment 2, sea cucumber was placed in the same location as experiment 1 in the experimental tank (Figure 1B). Sea cucumber was gently placed in the experimental tank without substrate or water flow (length × width × height: 660 × 410 × 250 mm, Figure 1A). A camera (Legria HF20; Canon, Tokyo, Japan) above the experimental tank recorded the movement of sea cucumbers, and the recording stopped when the sea cucumber reached the food zone. Each experiment was repeated 10 times using different sea cucumbers (N = 10). Seawater was changed for each test to avoid potential non-experimental impacts. Movement speed (*v*), distance (*l*), displacement (*d*), swing angle (*R*), and swing speed (*v_s_*) of sea cucumbers were calculated via Formulas (1)–(4).

### 2.3. Statistical Analysis

The data were tested for homogeneity of variance and normal distribution before all statistical analyses using the Levene test and Shapiro–Wilk test, respectively. Kruskal–Wallis *H* test was used to compare the duration, movement speed, and displacement among three states of sea cucumbers, because the data were non-normal and/or lacked homogeneity in the variance. Direction deviation, movement linearity, swing angle, and swing speed of sea cucumbers were compared using Mann–Whitney *U* test between the two experiments, because the data were non-normal and/or lacked homogeneity in the variance. All data analyses were performed using SPSS 25.0 statistical software. A probability level of *p* < 0.05 was considered significant.

## 3. Results

### 3.1. Experiment 1: Movement Pattern of A. japonicus without Food Cue

**Figure 2:**
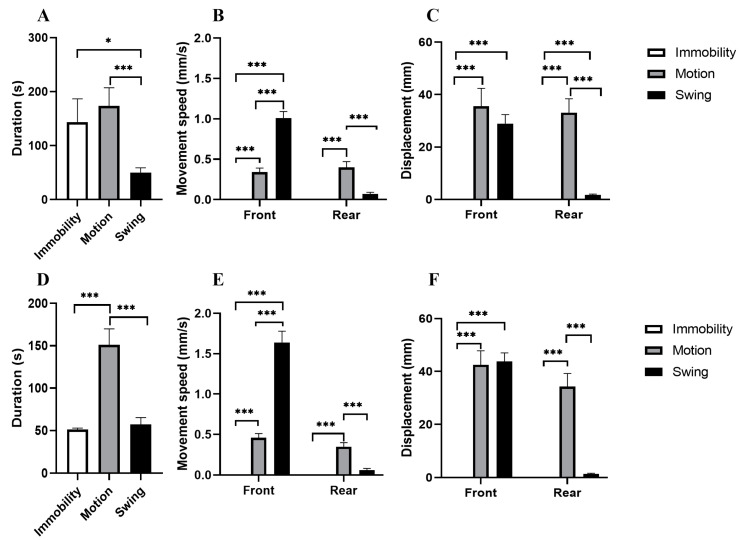
Duration of three states (**A**), movement speed (**B**), and displacement (**C**) of sea cucumbers in experiment 1. Duration of three states (**D**), movement speed (**E**), and displacement (**F**) of sea cucumbers in experiment 2. The asterisks * mean *p* < 0.05; The asterisks *** mean *p* < 0.001.

In experiment 1, a total of 13 immobility states (*n* = 13), 27 motion states (*n* = 27), and 49 swing states (*n* = 49) of sea cucumbers were recorded. The durations of these three states were 157.69 ± 155.46 s, 168.70 ± 174.90 s, and 48.98 ± 69.71 s, respectively (Figure 2A). There was no significant difference in the average duration of immobility and motion states (*p* = 0.376; Figure 2A). However, the duration of the swing state was significantly shorter than that of the immobility state and motion states (*p* = 0.023; *p* < 0.001; Figure 2A).

In the immobility state, the movement speed and displacement of the front body and rear body were significantly lower than that of the motion state (all *p* < 0.001; Figure 2B,C).

In the swing state, the movement speed of the front body was significantly higher than that of sea cucumbers in the immobility state and motion state (all *p* < 0.001; Figure 2B). However, the movement speed of the rear body of sea cucumbers in the swing state was significantly lower than that of individuals in the motion state (*p* < 0.001; Figure 2B). There was no significant difference in movement speed of the rear body of sea cucumbers between the swing state and immobility state (*p* = 1.000; Figure 2B). The movement displacement of the front body of sea cucumbers in the swing state was significantly higher than that of those in the immobility state (*p* < 0.001; Figure 2C). The movement displacement of the rear body of sea cucumbers in the swing state was significantly lower than that of individuals in the immobility and motion states (all *p* < 0.001; Figure 2C).

### 3.2. Experiment 2: Movement Pattern of A. japonicus in Response to Food Cue

In experiment 2, we recorded a total of 21 immobility states (*n* = 21), 44 motion states (*n* = 44), and 83 swing states (*n* = 83) of sea cucumbers. The duration of the motion state of sea cucumbers was significantly longer than that of the immobility state and the swing state (all *p* < 0.001; Figure 2D). There was no significant difference in the average duration between the immobility and swing states (*p* = 1.000; Figure 2D). 

In the immobility state, the movement speed and displacement of front body (all *p* < 0.001) and rear body (all *p* < 0.001) were significantly lower than that of the motion state (Figure 2E,F).

**Figure 3:**
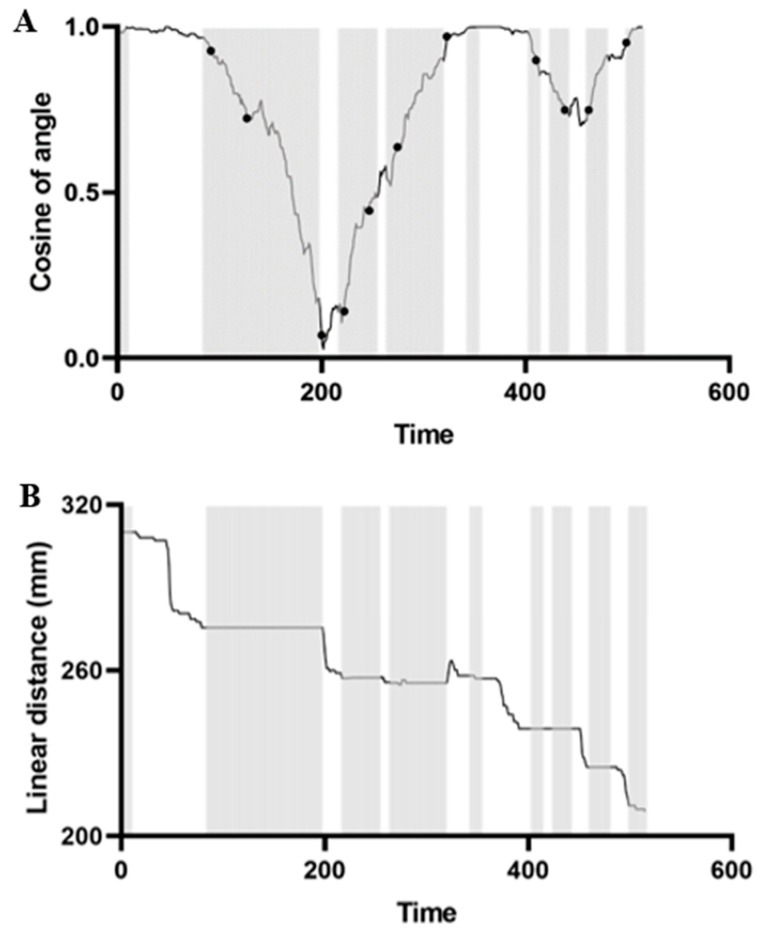
Change in angle cosine between the front of sea cucumber and the center of food area during the foraging movement. The gray area represents the sea cucumber in the swing state, and the black solid point is the segmentation point of broken line (**A**). The linear distance represents the distance between sea cucumber and the center of food cue zone. The gray area represents that the sea cucumber is in the state of swing (**B**).

The movement speed of the front body in the swing state was significantly higher than that in the stationary state and the motion state (all *p* < 0.001; Figure 2E). The movement speed of the rear body of sea cucumbers in the swing state was significantly lower than that of individuals in the motion state (*p* < 0.001; Figure 2E). There was no significant difference in the movement speed of the rear body of sea cucumbers in the swing state and the immobility state (*p* = 0.856; Figure 2E). The movement displacement of the front body of sea cucumbers in the swing state was significantly higher than that of those in the immobility state (*p* < 0.001; Figure 2F). The movement displacement of the rear body of sea cucumbers in the swing state was significantly lower than that of individuals in the motion state (*p* < 0.001; Figure 2F). The angle between the front body of the sea cucumber and the center of the food zone changed the most in the swing state (Figure 3A), and the straight line distance between the sea cucumber and the center of the food zone rarely changes (Figure 3B).

**Figure 4:**
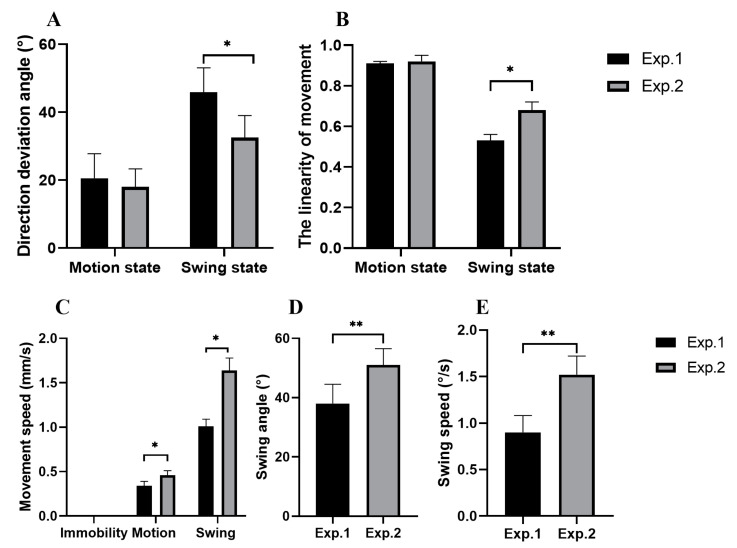
The movement direction deviation angle of sea cucumbers was affected by motion state and swing state (**A**). The movement linearity in motion state and after swing state (**B**). Movement speed of the front body of sea cucumbers in experiments 1 and 2 (**C**). Swing angle of sea cucumbers in experiments 1 and 2 (**D**). Swing speed of sea cucumbers in experiments 1 and 2 (**E**). The asterisks * mean *p* < 0.05; The asterisks ** mean *p* < 0.01.

There was no significant difference in the direction deviation angle of sea cucumbers in the motion state between experiments 1 and 2 (*p* = 0.194; Figure 4A). When sea cucumbers were in the swing state, the direction deviation angle in experiment 1 was significantly higher than that of those in experiment 2 (*p* = 0.038; Figure 4A). There was no significant difference in the movement linearity of sea cucumbers in the motion state between experiments 1 and 2 (*p* = 0.818; Figure 4B). The presence of food significantly increased the movement linearity of sea cucumbers in the swing state (*p* = 0.012; Figure 4B). Sea cucumbers exposed to the food significantly increased the movement speed of the front body in swing and motion states (*p* = 0.042; *p* = 0.017; Figure 4C). The swing angle and speed of the foraging movement pattern of sea cucumbers in experiment 2 were significantly higher than that of those in experiment 1 (*p* = 0.002; *p* = 0.008; Figure 4D,E).

## 4. Discussion

### 4.1. Swing but Not Motion Status Regulates the Direction of Movement When Sea Cucumbers Detect Food Cue

We revealed the characteristics of immobility, motion, and swing states in the movement mode by quantifying the movement behavior of sea cucumbers. These three states can be clearly distinguished by the speed of body movement in the front and rear of the sea cucumber. In the motion state, the front and rear movements of sea cucumbers were obvious, and the front movement speed was significantly higher. The contraction from the back to the front to produce displacement is consistent with the crawling behavior of sea cucumbers [31]. We found that the motion linearity of sea cucumbers is 0.91 ± 0.01 in the motion state, indicating that the motion state of sea cucumbers is close to linear. In addition, the effect of the motion state on the deviation angle of motion direction is only about 20 degrees. This further indicates that the change of motion direction is very small when sea cucumbers are in the motion state. The present study not only revealed that the movement state was the main state of motor function, but also found that the movement direction of sea cucumbers in motion state was close to linear motion. Previous studies have noted that the movement of sea cucumbers is random in situations where food is excessively abundant and impoverished [16,23,32]. Therefore, we speculate that there should be other behaviors involved in changing the movement direction of sea cucumbers. To further investigate the mechanism of regulating the direction of movement, we conducted a foraging behavior experiment on sea cucumbers in the presence of food cues. In the foraging experiment, the movement pattern is consistently composed of immobility, motion, and swing. The angle cosine value between the body and the center of the food area frequently changes in the foraging process. Interestingly, these changes mainly occur in the swing state, with minimal changes in immobility and motion states. These characteristics indicate that swing is the state in which sea cucumbers most frequently adjust the direction of movement, making sea cucumbers accurately move toward food. This is different from the swing behavior of snails *Helix pomatia*, which connect with their partners by swinging their heads during the courtship [33]. Mantis *Hierodula crassa* exhibits slight body swings during predation [34]. The swing behavior of sea cucumbers is similar to that of some invertebrates. Leech *Hirudo medicinalis* pushes its body forward through the free bending of its body, while the swing behavior affects the direction of movement [35]. *Caenorhabditis elegans* change their navigational behavior by swinging their heads [36]. Collectively, the swing state is an important state in the motion mode for sea cucumbers to regulate the movement direction, and the motion state does not change the direction of movement. This movement pattern, in which the body moves forward in the motion state and adjusts the direction of movement in the swing state, has great application potential in biomimetics [37]. The present study provides valuable insights into the movement of eyeless organisms. Notably, the present study only describes the movement patterns of sea cucumbers when they detect food cues. Sea cucumbers live in a complex environment, and the present study is a laboratory experiment that does not take into account the effects of other factors, including predators, individual size, and so on.

### 4.2. Movement Is Not Random but Directional in Sea Cucumbers Exposed to Food Cue

We further found that the immobility state time significantly reduced compared with that in experiment 1. The frequency of motion state response to food was 1.6 times higher than that in experiment 1. This indicates that food cue causes the change in motion state. Fewer stays and more frequent movement over short periods of time are viable for animals to find food nearby during foraging [38,39]. In addition, all sea cucumbers finally reached the location of the food cue in the present foraging experiment. This indicates that the response of sea cucumbers to food is not completely random but is directional toward food. This is in contrast to previous reports that the foraging movement of sea cucumbers is a random walk model [27]. The disagreement is probably because of the different densities of food cues. In the random motion model experiment, sufficient food cue was evenly dispersed in the experimental container, so sea cucumbers could have rapid access to the food cue. This experimental design is not considered a good representation of the overall foraging behavior of sea cucumbers. In this study, the food cue was, therefore, set at a distance from the sea cucumber. Collectively, foraging behavior experiments showed that food cues caused the changes in the movement state of sea cucumbers and that the movement in the presence of food was directed rather than random.

### 4.3. Food Cue Significantly Affects the Swing Behavior of Sea Cucumbers

Food cues affect the foraging and feeding behaviors of sea cucumbers. Interestingly, food cues significantly affected the swing behavior. The frequency of the swing state in experiment 2 was 1.7 times higher than that in experiment 1. The swing angle and swing speed of sea cucumbers were significantly higher in experiment 2 than those in experiment 1. The motion of the sea cucumbers is consistent with the exploratory activity in experiment 1. However, swing behavior was more frequent in experiment 2. We suspect that sea cucumbers, as eyeless organisms, receive food cues via chemo-sensing in foraging behavior [40], acquire cues via frequent swings to maintain a sense of direction, and adjust the direction of movement in time. This result was similar to the head swing behavior of the slug *Limax marginatus*, which showed a strong head swing near the odor source [41]. Combined with previous studies, we further suggest that the swing behavior plays two functions in the foraging of sea cucumbers. First, swing behavior adjusts the direction of movement to make sea cucumbers to move to the food position. More significant changes in the direction of movement can ensure that sea cucumbers stay and explore for a long time. Second, swing behavior is an exploratory activity. Collectively, the foraging behavior experiment showed that food cues caused changes in the movement state of sea cucumbers and that the movement in the presence of food was not random but directed in sea cucumbers.

## 5. Conclusions

The motion state of sea cucumbers was close to a linear motion. Sea cucumbers adjusted the direction of movement most frequently in the swing state. The motion state did not change the direction of movement, while the swing state regulated the direction of movement. In the foraging experiment, the immobility state of sea cucumbers was significantly lower in the presence of food cue than that of those in the absence of food cue, while the motion state response to food cue was frequently higher than that of those without food cue. Swinging frequency, swinging angle, and swinging velocity were significantly higher in experiment 2 than those in experiment 1. These results suggest that food cues not only cause the change in the motion state but significantly influence the swing behavior of sea cucumbers. In addition, the foraging experiment also found that the movement pattern of sea cucumbers did not fully conform to the random walk model but a directional movement to food cue. These novel findings complement the study of movement and foraging behaviors of sea cucumbers and provide valuable information into the behavioral mechanism of regulating movement direction in eyeless organisms. Notably, movement patterns are probably more complex than we described because a number of environmental factors (for example, predators) are not involved in the present study.

## Data Availability

All of the data generated or analyzed during this study are included in this published article.
